# The Influence of Residual Stresses on the Curve Shape—Describing Interface Behavior in “Polymer–Fiber” Systems

**DOI:** 10.3390/polym16050582

**Published:** 2024-02-21

**Authors:** Yulia A. Gorbatkina, Viktoria G. Ivanova-Mumzhieva, Olga V. Alexeeva, Mariya A. Vyatkina

**Affiliations:** 1 N.N. Semenov Federal Research Center for Chemical Physics, Russian Academy of Sciences, 119991 Moscow, Russia; tamiku.ain@yandex.ru; 2 N.M. Emanuel Institute of Biochemical Physics, Russian Academy of Sciences, 119334 Moscow, Russia; alexol@yandex.ru

**Keywords:** adhesive strength, polymer–fiber bond, pull-out method, residual stresses, curing process, temperature tests

## Abstract

The pull-out method was used to study the adhesive strength τ of “fiber–thermoset” systems with wide variations in area. Studied binders were based on resins that had different chemical natures (epoxy, epoxy phenol, orthophthalic, polyphenylsiloxane, and phenol–formaldehyde). Shear adhesive strength was determined for systems with two fiber types (glass and steel fibers). It was shown that strength τ depended on scale (area). Formation of τ occurred during the curing process and the system’s subsequent cooling to the measurement temperature T. It was found that interface strength depended on measurement temperature across a wide temperature range that covered the highly elastic and the glassy state of the adhesive. The influence of residual stresses τ_res_, acting at the “binder–fiber” interface, on the nature of the curves describing the dependence of the adhesive strength on the studied factor was experimentally shown. A qualitative explanation of the observed regularities is proposed.

## 1. Introduction

In the middle of the last century, fibrous composite materials widely and actively entered our lives. It is well known, now, that the properties of these materials depend not only on the properties of the fibers and matrices, but also on the properties of the interphase boundary between them. Primarily, these properties depend on the adhesion strength of the fibers to the matrix [1,2,3,4,5,6,7,8,9,10,11,12,13,14,15,16,17,18,19,20].

The ability to use the high strength of thin fibrous reinforcing fillers under various types of external influences appears to be due to the adhesive strength at which external loads are transferred to the fibers [21,22,23,24,25,26,27].

The fiber, together with the adjacent layer of polymer adhesive, constitute the elementary unit of any fiber composite. As a rule, models of such elementary units are used in experiments to measure the strength of polymers with fibers. In most cases, the fiber is pulled out of the cured matrix layer (pull-out method), determining the shear adhesive strength τ, which is also called the interface strength. Adhesion to “thick” fibers with a diameter d greater than 100 μm was first determined in 1959 [28], and to “thin” fibers with d = 10–20 μm, in 1962 [29]. Thus, shear adhesive strength in polymer–fiber systems has been measured for more than 60 years. Both of these works used different variations of the pull-out method. Since then, measurement techniques and result processing have been improved and developed [15,30,31,32,33,34,35,36,37,38,39]. However, the measurement technique still cannot be considered fully developed, since there is a huge variety of adhesive pairs with conditions for manufacturing adhesive joints that cannot be regulated. This is also associated with a number of changing conditions: the nature of adhesives and their various initial states, such as liquids of various viscosities, granules, powders, films, and particles of various shapes and sizes, including nano-sized ones. The nature of the substrates (fibers) and their diameters, as well as the morphology of their surfaces and different heat treatment modes when creating compounds have a great influence.

**Figure 1:**
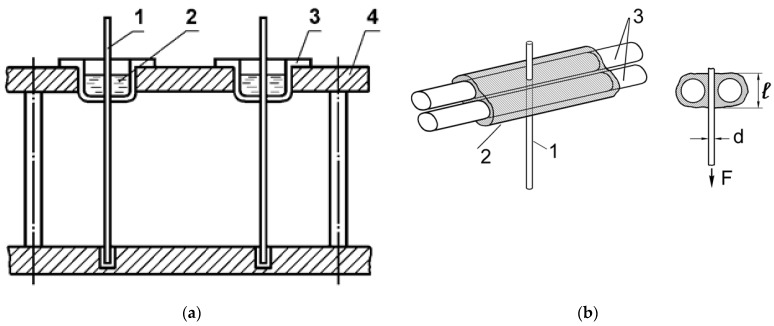
Schemes of samples used to determine the adhesive strength of polymer–fiber compounds using the pull-out method. (**a**) Substrate is fiber with diameter d ≥ 80 μm according to the “classical method”: 1—fiber, 2—polymer, 3—aluminum cup, 4—device for preparing samples; (**b**) substrate is fiber with d ≈ 10–13 μm according to the “three fiber method”: 1—“thin” fiber with a diameter d, adhesion to which is determined, 2—a polymer layer of length l, 3—two thick fibers—“carriers” of the resin.

Summarizing the above, we can conclude that a large number of factors affecting adhesive strength are eliminated by the creation of standards regulating the conditions for producing and testing polymer–fiber joints. For fibers of different diameters, different methods are used to prepare samples suitable for reliable determination of adhesive strength. The simplest diagrams of the samples are shown in Figure 1.

The adhesive strength of samples is calculated using the formula:

(1) $$\tau =\frac{F}{S}=\frac{F}{\pi dl}$$

where F is the force required to shear the fiber over the adhesive layer, d is the fiber diameter, l is the length of the adhesive joint (the length of the fiber section immersed in the resin), and S is the area of the adhesive joint (the contact area of the adhesive and the fiber). In experiments, force F and length l are measured. The values of F and l are used to calculate the adhesive strength and its dispersion.

The value of the interface strength calculated using Formula (1) is very arbitrary. The exact execution of Formula (1) assumes the following:

- Round fiber cross-section.
- Constant diameter of the fiber immersed in resin.
- Good wetting of the fiber by the binder, i.e., the absence of any discontinuities in the area of the fiber immersed in the binder, where the adhesive joint is formed. And, accordingly, the equality of the visible (measured when determining the adhesive strength) and the true area of contact between the fiber and the adhesive.
- Uniform distribution of tangential stresses acting at the “adhesive–fiber” interface.

The last assumption for connections with an interface is not satisfied if measurements of τ are carried out below the glass transition temperature of the adhesive.

Due to the differences in thermal and mechanical characteristics of the adhesive and the substrate, such as the coefficient of linear expansion, elastic modulus, and Poisson’s ratio, residual stresses arise at the interface. They are unevenly distributed along the length of the joint (gluing length): they are maximum at the ends of the joint, pass through “zero” in the middle of the joint, and increase almost linearly (to a first approximation) with decreasing temperature. Residual stresses are formed during the curing of the adhesive joint and its further cooling to the test temperature. They exist at the interface before any application of external force. When a load is applied to the compound, they add up to the stresses arising from the application of an external force, which is also unevenly distributed.

In polymer–fiber compounds, the value of residual stress cannot be measured directly. Methods for such measurements have not yet been created. However, the presence of these stresses is revealed by experimental study of the dependence of adhesive strength on the action of various external factors.

Since the values of residual stresses depend on the joint area S, the general patterns of their influence on the measured values of adhesive strength can be established when measuring τ in a wide range of S values. As will be shown below, this condition may not always be satisfied. Apparently, therefore, the role of residual stresses in discussing the results of measurements of the interface strength and fracture mechanisms in polymer–fiber systems is not always analyzed.

In this work, using examples, we experimentally showed the influence of residual stresses acting at the interface in polymer–fiber systems on the form of curves describing the change in shear adhesive strength depending on the factor under study. Particular attention was paid to the study of systems in which the length of the gluing could be widely varied.

We carried out a comprehensive study of polymer–fiber adhesive systems as an elementary unit of reinforced plastic to allow us to assess the contribution of residual stresses to the strength of such materials. This study will also help determine possible mechanisms of their destruction and identify the weakest element in the structure of reinforced plastics. The described experimental approach will be useful in modeling the processes of fracture of reinforced plastics and searching for ways to increase their elastic-strength properties, including through the organization of more effective interactions at the polymer–fiber interface.

## 2. Materials

In this work, several binders, with different chemical compositions, that are used for the manufacture of reinforced plastics, were studied: EDT-10, EAnhB, MAB, PPSR, BP-4, NP-1, and 5-211. The EDT-10 binder was a mixture of epoxy resin ED-20 (JSC CHIMEX Limited, Moscow, Russia), an active diluent of diethylene glycol diglycidyl ether DEG-1 (CHIMEX Limited, Russia), and a triethanolamino-titanate hardener (TEAT, JSC CHIMEX Limited, Russia) with the component ratio of 83.4:8.3:8.3 wt.%. Epoxyanhydride adhesive (EAnhB) was a mixture of epoxy resin ED-20, isomethyltetrahydrophatelic anhydride hardener (JSC CHIMEX Limited, Russia), and 2-methylimidazole accelerator (JSC CHIMEX Limited, Russia) in a ratio of 52.6:47.3:0.1 wt.%. The modified epoxyamine binder (MAB) consisted of ED-20, DEG-1, TEAT in a ratio of 77.0:7.7:15.4 wt.%. We also used poly methyl phenyl siloxane resin PPSR (Khimprom, Novocheboksarsk, Russia), butvaro-phenolic glue BP-4 (Solins, Guangzhou, China), and epoxyphenol binder grade 5-211 (NRC “Kurchatov Institute” VIAM, Moscow, Russia). The PN-1 polyester resin contained PN-1 orthophthalic resin (JSC Raduga-Sintez, Elektrougli, Russia) and Butanox M50 hardener (methyl ethyl ketone peroxide in a phthalic-based plasticizer) (Azkonobel, Vilvoorde, Belgium) in a ratio of 98.8:1.2 vol. %.

In the process of preparing samples, the substrate for determining the adhesive strength of polymer–fiber systems was alkali-free glass fibers with a diameter of 10–13 μm, which are destroyed under a load of 0.3–0.5 N; tensile strength 3500 MPa (JSC NPO Stekloplastic, Solnechnogorsk, Russia). Steel fibers with a diameter of 150 μm (OVS steel wire with a tensile strength of 2800 MPa) were also used.

## 3. Methods

Diagrams of samples used to manufacture systems with fibers of various diameters are shown in Figure 1. All samples were tested on adhesiometers (microfracture machines) developed in the Laboratory of Reinforced Plastics FRC CP RAS [6]. Measurement error was 8–10%. The methods for preparing samples and processing the results were described in detail earlier [6]. Differential scanning calorimetry (DSC) (NETZSCH DSC 204 F1 Phoenix, NETZSCH-Gerätebau GmbH, Selb, Germany) was used to determine the thermal effects of the curing process of modified binders and the glass transition temperature of matrices in the measurement temperature range of 25–250 °C, with a heating rate of 10 °C·min^−1^ under argon. Samples were heated twice. From the obtained DSC curve for the first heating, the heat of the curing reaction was determined, and from the second, the glass transition temperature T_g_ of the samples. The degree of binder curing was calculated using the formula:

(2) $$\alpha =(1-\frac{H_{S}}{H_{T}})\cdot 100\%$$

where α is curing degree, %; H_S_ is total heat of reaction of partially cured binder, J·g^−1^; and H_T_ is total heat of reaction of a fully cured binder, J·g^−^^1^. The error in measuring the heat of transformation was 3%, the error in determining T_g_ was ± 3 °C.

## 4. Results and Discussion

### 4.1. The Influence of Residual Stresses on the Dependences F–S and τ–S (Scale Dependence of Adhesive Strength)

As mentioned above, the interface reacted to external factors. This can be concluded from the measured force required for adhesive fracture of systems of length l.

**Figure 2:**
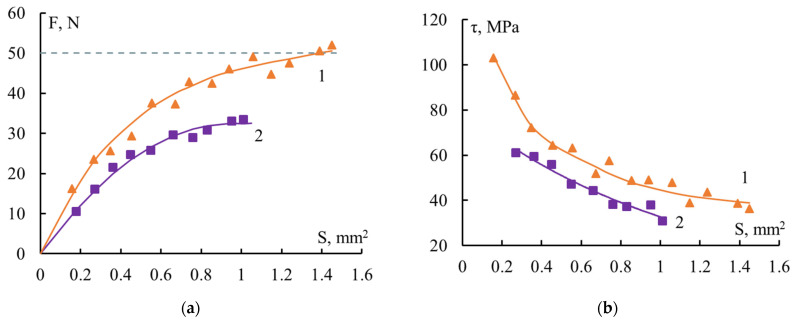

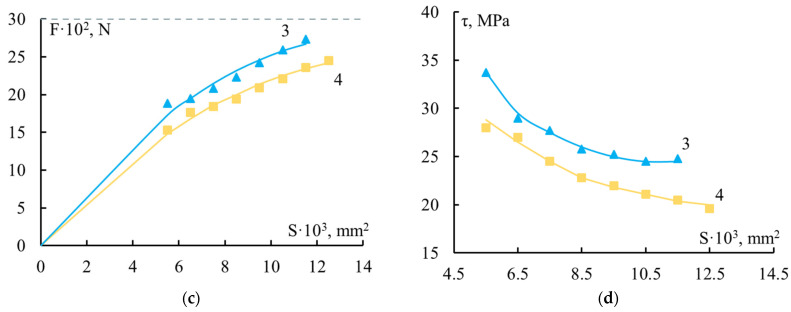
Dependence of the force F required to shear the fiber versus the binder layer (**a**,**c**) and the adhesive strength (**b**,**d**) of the fiber–binder systems versus the bonding area S. Fibers: steel, d = 150 μm (**a**,**b**); alkali-free glass d ≈ 13 μm (**c**,**d**). Binders: 1—EDT-10, 2—EanhB, 3—MAB, 4—PPRS. When calculating the values of τ (Figure 2 (**c**,**d**)), only the strengths of samples that adhesively broke along the interface were used. The dotted lines indicates fiber strength. The compositions of the binders are presented in Table 1.

Figure 2 shows the F–S and τ–S dependences typical of fiber–thermoset binder systems if τ measurements, obtained from these data, were taken below the glass transition region of the adhesive.

From the shapes of the curves it can be concluded that when both thin glass fibers and much thicker wires were used as substrates, the adhesive strength changed quite noticeably as the size of the systems changed. With increasing S, the value of F increased and then reached a constant level. The maximum value of the force F was determined by the strength of the fibers to which adhesion was measured. The F values at which the steel wire and glass fiber failed are marked with a dotted line in Figure 2a,c. The values of τ monotonically and nonlinearly decreased. The most noticeable change in values was observed at small S. The larger the range of areas where the adhesive strength was determined, the more noticeable the dependence of τ on S. This means that the influence of the scale factor was more noticeable.

Table 2 shows the ranges of changes in the areas and lengths of the systems under study (S and l). Also given are the values of the dimensionless parameter l/d, which is convenient to use when comparing the adhesive strength of systems with fibers of different diameters. It can be seen that when thin industrial glass fibers were used as a substrate, the range of changes in S was small. The S values differed by no more than 2–3 times. This is explained by the fact that fibers with a diameter d ≈ 7–20 μm cannot be spliced with a length l less than 150–200 μm, that is, for them the minimum l/d ratio is always greater than ten. For the adhesion of polymers to steel wire (and other high-strength fibers) with a diameter d > 80–100 μm, the minimum value of l/d is several units. Accordingly, the measurement range S was much wider. The S_max_/S_min_ ratio was 7.5 or more. Therefore, it was necessary to use relatively high-strength fibers with high tensile strength to identify general patterns of changes in interface strength in polymer–fiber systems. Only in this case could a wide variation of the joint areas be achieved. When studying the influence of the same factor in “polymer–thin fiber” systems on the mechanism of fracture, the patterns of influence on bonding at l/d < 10 could not be established.

The dependence of F and τ on the joint area S makes it difficult to compare the adhesive strength of different pairs, since it is difficult to make a comparison “all other things being equal”. So, for example, in a comparative assessment of the adhesion of different resins of the same nature to a fiber of constant diameter, as in Figure 2, the adhesive ability of the binders could be judged by the relative position of the curves: “more–less”. This method of comparison has been used in a variety of studies [6].

In this case, the form of the curves describing the dependences τ = τ(S), τ = τ(l), and τ = τ(l/d) is the same [33].

Curves similar to those shown in Figure 2 are observed for various binders: epoxy, modified epoxy, polyester, and phenol–formaldehyde, as well as for ladder and linear polymers, etc., when interacting with various fibers (metal, glass, carbon, basalt, silicon carbide, textile, etc.), of completely different diameters, provided that the measurements are carried out at temperatures below the glass transition temperature of the binder [39].

If the adhesive is in a glassed state, the values of F and τ are functions of the bonding area. With all the variety of adhesion pairs, the appearance of the F–S and τ–S curves corresponding to different pairs was the same (see Figure 2). This, first of all, depends on the fact that the physical laws of the formation of residual stresses are the same for all systems. And the magnitude of residual stresses is different for different pairs since they use adhesives and substrates with different properties. Accordingly, the values of τ are different for different pairs.

The values of τ may not depend on the area of joints S in two cases:

1. If measurements of τ are carried out in the glass transition region of the adhesive or at temperatures above it. This is observed, for example, when studying adhesion in polyolefin-fiber systems at room temperature. Residual stresses under these conditions are either zero or can relax.
2. If measurements of τ are carried out in the glass transition region of the adhesive, but in a very narrow range of areas, especially if this interval corresponds to large values of S (l/d ≥ 7–10). Residual stresses in a narrow interval S change little and, accordingly, changes in the values of τ are difficult to notice since they lie within the limits of measurement errors.

The appearance of the τ–S curves in Figure 2 (no matter what the underlying causes of the dependence of τ on S) suggests that the value of adhesive strength measured in experiments cannot characterize the adhesion in a given pair, since F and τ are functions of the dimensions of the system.

Thus, the measure of adhesion in a given pair can be the value of τ as S tends to zero. This value was called the local adhesive strength τ_loc_ [6].

(3) $$\tau_{loc}=\underset{S\to 0}{\lim}\frac{F}{S}=\underset{S\to 0}{\lim}\tau (S),$$

In [6], at a qualitative level, using a simple shear analysis (using the Cox method [40] in the form given to it by Kelly [41]), the law of extrapolation of τ–S (S → 0) curves was derived and its application was shown. It is quite difficult to determine the value of τ_loc_ with good accuracy. To do this, you need to measure the τ–S dependence in a wide range of gluing. Then, extrapolation to zero can be carried out with acceptable accuracy.

Experience shows that, as a first approximation, the value of τ_loc_ can be taken to be τ at l/d~2–3. When using steel wire with a diameter of 150 μm as a substrate (as in many of our experiments), this corresponds to l = 300–500 μm or S = 0.2–0.25 mm^2^.

In [6], it was also proposed to use the value of τ_loc_ when formulating the conditions for fracture of the polymer–fiber systems under consideration.

Adhesive fracture of polymer–fiber systems occurs when the sum of external and residual stresses acting at the interface is equal to the local adhesive strength.

(4) $$\tau_{exp}+\tau_{res}=\tau_{loc}$$

All results given below were obtained based on this equation.

When studying how τ changes under the influence of one or another factor, it often becomes necessary to compare the change in τ with the change in the properties of the binder (adhesive) and (or) the properties of the composites. This comparison must take place under the same conditions and be derived from the same components as the adhesive system. Based on the above results, it can be concluded that to construct correlation curves of “binder properties–adhesive strength”, values of τ that are free from the action of residual stresses should be used, namely the values of τ_loc_ or the values of τ for small gluing areas. Since in a homogeneous binder there are no residual stresses, there are, accordingly, no such effect on properties. To obtain correlation curves of the “fiber composite properties–adhesive strength” type, τ values should be used for large gluing areas because, in an elementary unit of a composite material, the sum of all stresses—external and residual—acts.

As a result, when studying the scale dependence of the adhesive strength of the polymer–fiber system, the uneven distribution of residual stresses (primarily temperature) led to a change in the nature of the curve of dependence of τ on S. Now, it was described not by a straight line parallel to the x-axis, but by a monotonically nonlinearly decreasing curve.

This led us to the need to introduce the concept of local adhesive strength and allowed us to formulate at a qualitative level, the conditions for the destruction of the polymer–fiber system.

### 4.2. Influence of Residual Stresses on the Dependences τ–t_cur_ (Adhesive Strength–Curing Time)

Now we will consider the influence of residual stresses on the types of curves that describe the formation of adhesive strength during the curing “fiber–thermosetting binder” of adhesive systems.

**Figure 3:**
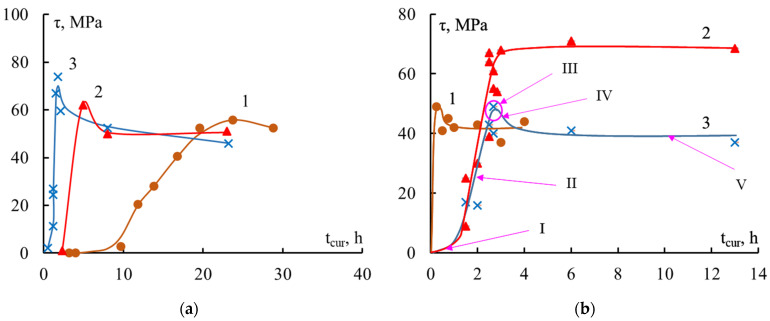
Change in adhesive strength τ of “steel fiber–epoxy binder” systems during the curing process. (**a**) EDT-10 binder, isothermal curing at 1—80 °C; 2—120 °C; 3—160 °C; S = 0.55 mm^2^ and (**b**) EAnhB binder, isothermal curing at 1—140 °C, S = 0.55 mm^2^; non-isothermal curing: 3 h at 90 °C and 12 h at 120 °C; 2—S = 0.25 mm^2^; 3—S = 0.65 mm^2^. Composition of the binders, see Table 1; curing sections I–V, see text.

The dependences in Figure 3 show the kinetics of the formation of adhesive strength in the “steel fiber–epoxy binder” system. It can be seen that the strength of the interface did not appear instantly, but developed gradually. The dependence τ–t_cur_ was clearly divided into several sections, that is, the formation process took place in several stages.

**Figure 4:**
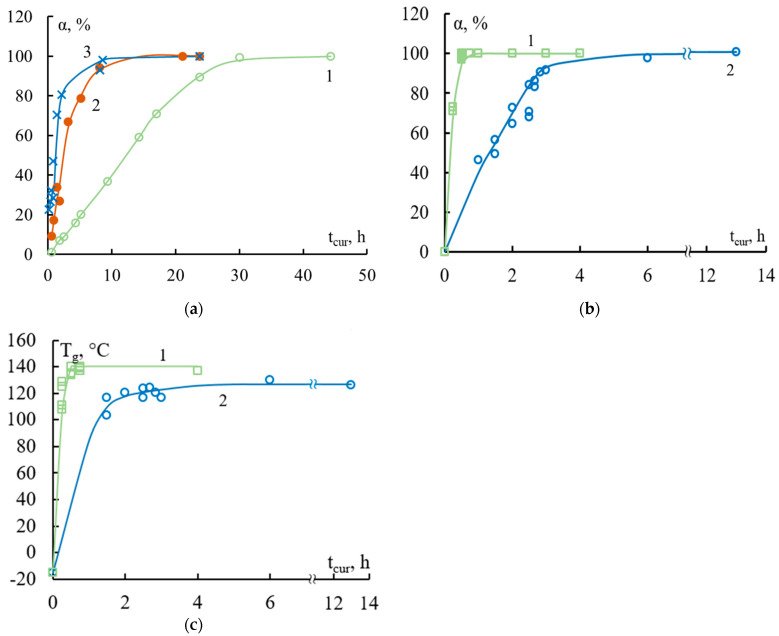
The influence of temperature and time conditions for the formation of “epoxy binder—steel fiber” adhesive systems with d = 150 μm on the degree of curing α and the glass transition temperature T_g_. Binders: (**a**) EDT-10, isothermal curing at temperatures 1—80 °C, 2—120 °C, 3—160 °C; (**b**,**c**) EAnhB, 1—isothermal curing at 140 °C; 2—non-isothermal curing 90 °C for three hours + 120 °C for twelve hours. For composition of the binders, see Table 1.

The first section (I, Figure 3b) corresponded to the beginning of the curing process—this was the induction period. Here the binder was a viscous liquid, fracture occurred cohesively along the binder, and the adhesive strength was practically zero. The question of whether it really was zero or could not be measured was discussed earlier in [6]. In the second section (II, Figure 3b), a mesh structure of the adhesive was formed. Network nodes were formed, the glass transition temperature T_g_ increased, and viscosity quickly increased. At the end of this stage, the binder passed the gel point, after which the formation of new nodes practically stopped due to the very high viscosity. As the degree of curing increased (see Figure 4), the physical and mechanical properties of the epoxy adhesive were formed, adhesive bonds arose at the polymer–fiber interface, and the adhesive strength increased. The kinetics of bond formation in the bulk and at the interface were not the same: there was neither a maximum nor an induction period on the α–t_cur_ and T_g_–t_cur_ curves (Figure 4).

This is well demonstrated by the data in Figure 3 and Figure 4, which show the curves τ–t_cur_, T_g_–t_cur_, and α–t_cur_. During the curing process of the studied binders, bonds are formed in the volume and volumetric properties arise that determine their adhesive strength in joints with fibers. For epoxyamine and epoxyanhydride binders, the given dependences were typical.

The values of τ could be measured with great reliability when the degree of curing α reached 55–65%. At these values of α, it can be assumed that the effect of residual stresses also becomes noticeable: the rate of growth of the interface strength τ slows down. At α ≥ 60–70%, the F–S and τ–S dependences took on typical form of polymer–fiber systems below the glass transition temperature of the adhesive. This was detailed in the previous section in Figure 2.

As shown above, in the second section of the τ–t_cur_ curve there were two processes; the speed of both changed over time:

1. The interface strength was formed.
2. The adhesive structure was formed.

The combined action of these processes led to the emergence and growth of residual stresses, which caused a decrease in the adhesive strength values measured experimentally (in accordance with Formula (4)). As a result, a maximum appeared on the τ–t_cur_ curve.

The third section (III, Figure 3b) was the maximum zone on the τ–t_cur_ curve. After the maximum, the values of τ decreased slightly (IV section), and then remained unchanged throughout the rest of the heating of the system (V section).

An increase in the size of the sample and the contact area S led to an increase in residual stresses and, as a consequence, there should have been a greater decrease in the adhesive strength after the maximum. As can be seen in Figure 3b, the experiment confirmed this conclusion: at S = 0.2 mm^2^ there was no maximum on the curve, but at S = 0.65 mm^2^ it was quite clearly visible.

Thus, not only the values of the adhesive strength on the τ–t_cur_ curve, but also the appearance of the τ–t_cur_ curve itself, which describes the behavior of the interfacial strength, depended on the size of the sample.

It should be noted that all stages of formation of adhesive strength were clearly observed during isothermal curing. In a non-isothermal process, individual sections on the τ–t_cur_ curves may be completely absent. It all depends on how the temperature-time regime of the curing process is selected.

Thus, the type of curve describing the formation of the interface in “thermosetting binder–fiber” systems, τ–t_cur_, depends not only on the selected curing mode, but also on the dimensions of the sample. This is determined by the dependence of the magnitude of residual stresses acting at the interface on the dimensions of the gluing place. In the general case, the τ–t_cur_ curve has a maximum, which should be more strongly expressed as the gluing size S increases. If the gluing size is small (l/d ≤ 4), the maximum may be completely absent. Such curves have been observed for systems based on various amine-cured epoxy resins [6] and anhydride-cured epoxy resin ED-20 [42].

### 4.3. Influence of Residual Stresses on the Dependences τ–T (Adhesive Strength–Test Temperature)

The third series of experiments was the study of temperature dependences with wide variations in the area of joints. Here the influence of residual stresses on the behavior of the interface between polymer and fiber systems was clearly demonstrated.

**Figure 5:**
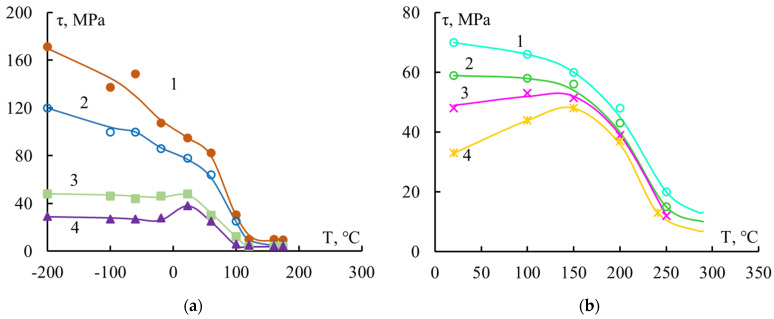
Dependence of the adhesive strength of epoxyamine binder systems with steel fibers with d = 150 μm versus experimental temperature. Binders: (**a**) EDT-10: 1—S = 0 (τ = τ_loc_); 2—S = 0.15 mm^2^; 3—S = 0.55 mm^2^; 4—S = 1.15 mm^2^; (**b**) MAB: 1—S = 0.35 mm^2^; 2—S = 0.45 mm^2^; 3—S = 0.55 mm^2^; 4—S = 0.75 mm^2^.

Figure 5 shows such dependencies for connections of two adhesive pairs. Each curve in Figure 5 is divided into three sections:

(1) Above the glass transition region. In this case, the binder was in a highly elastic state. The values of τ were very small and there was little dependence on the size of the junction.
(2) The glass transition region is the region where the binder transitions from a highly elastic to a solid (glassy) state. The values of τ increased monotonically with decreasing temperature and changed slightly as S changed.
(3) Below the glass transition region, the binder was in a solid state. In this temperature range, not only the values of τ, but also the appearance of the τ–T curves, depended on the area of contact between the resin and the fiber.

The observed appearance of the curves was determined by the residual stresses τ_res_ acting at the interface. Above the glass transition region, residual stresses can practically be ignored since they can relax. In the glass transition region, where the rigidity of the binder was still low, the residual stresses were small and did not change the course of the τ–T curves. Adhesive strength at any resin–fiber contact area increases rapidly with decreasing temperature.

Below the glass transition region, as mentioned earlier, residual stresses increase almost linearly with decreasing temperature. At a given temperature, the values of τ_res_ increase with increasing S.

In polymer–fiber systems, two processes develop in this region with decreasing temperature:

1. Local adhesive strength τ_loc_ increases;
2. Residual stresses τ_res_ increase.

**Figure 6:**
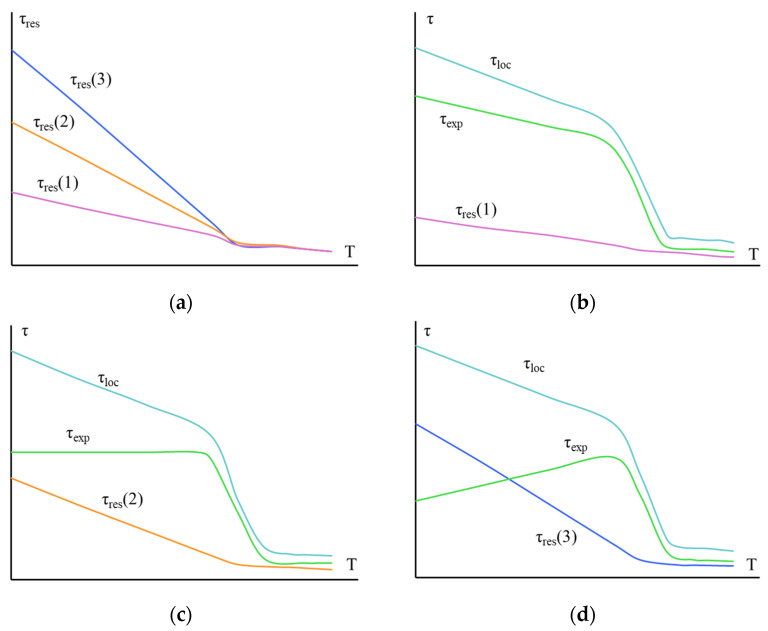
Diagram illustrating changes in test temperature and residual stress values τ_res_, local adhesive strength τ_loc_, and experimentally measured values of adhesive strength τ_exp_ of a polymer–fiber adhesive pair with different gluing areas S. S_1_ < S_2_ < S_3_ (**b**, **c** and **d** respectively); τ_exp_ = τ_loc_ − τ_res_.

The superposition of these processes on each other can lead to both monotonic and non-monotonic changes in the values of adhesive strength. The overlay of the mentioned processes is shown schematically in Figure 6.

Figure 6a shows how residual stresses τ_res_(1) < τ_res_(2) < τ_res_(3) increased in joints with area S_1_ < S_2_ < S_3_. Figure 6b–d demonstrate how a change in τ_res_ affected the experimentally measured values of adhesive strength τ of a given pair. When depicting, it was taken into account that τ = τ_loc_ – τ_res_ and that the local adhesive strength of a given pair was the same for samples with any area.

Thus, when measuring the adhesive strength of the same “polymer–fiber” adhesive pair in a wide temperature range with a wide variation in the area of gluing, three types of τ–T curves can be observed.

As temperature increases, strength can gradually decrease, stay the same first and then go down, or first increase, and then decrease.

All three types of τ–T curve can be observed if samples with a wide range of areas S (l/d~3—15–20) are used. The examples given (Figure 5) also show dependencies for systems where high-strength steel fibers were used for the substrate.

**Figure 7:**
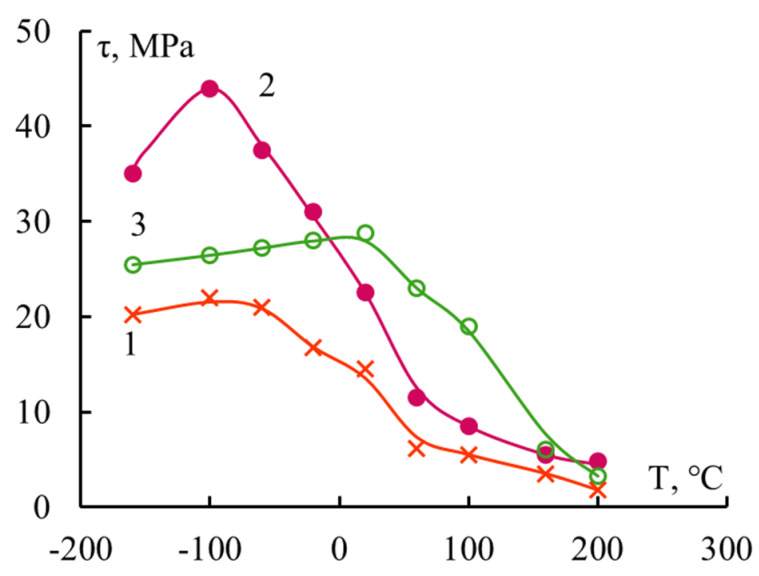
Adhesive strength of “thermosetting binder–alkali-free glass fibers systems with d ≈ 13 μm; binders: 1—polyester PN-1, 2—BP-4; 3—silicon–organic PPSR.

If the τ–T dependence is studied for polymer systems with “thin” fibers with a diameter d~7–20 μm (Table 3), then the range of changes in the l/d parameter turns out to be significantly narrower. As already mentioned, for these fibers it is almost impossible to prepare connections with a gluing length l less than 150–200 μm, that is, with l/d < 10–15. In accordance with the developed concepts, the τ–T dependence in these cases should be described by a curve with a maximum. This experiment shows the correctness of this statement. Several curves are shown in Figure 7. The nature of the adhesives in these pairs is different, and the strength of the interface in a wide temperature range for all pairs is described by a curve with a maximum.

**Figure 8:**
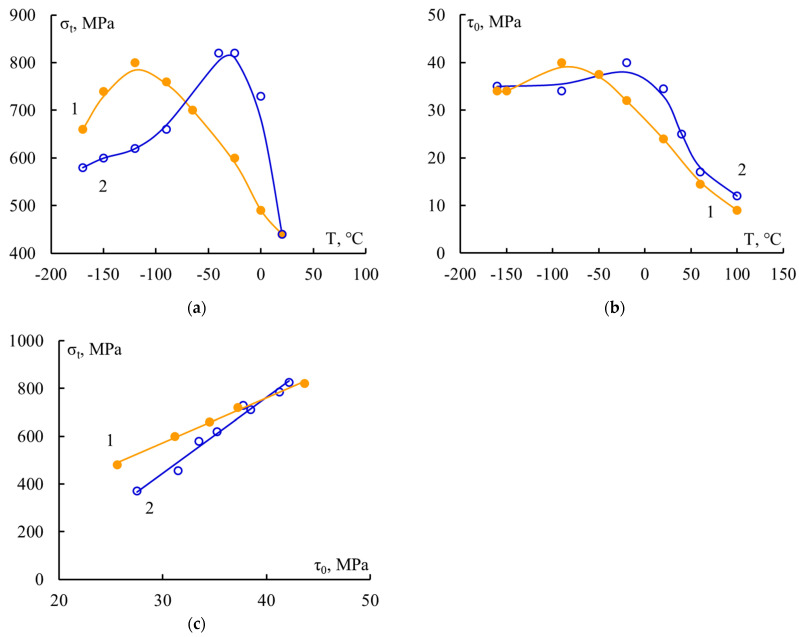
Dependence of the tensile strength of unidirectional fiberglass plastic (**a**) and the adhesive strength of “matrix-fiber” systems (**b**) versus test temperature; (**c**) correlation curves σ_t_–τ_0_; matrices: 1—BP-4, 2—5-211. τ_0_ = τ + ∆τ; τ is the arithmetic mean of the strength of samples that failed adhesively; ∆τ is the correction associated with taking into account the samples that failed cohesively. For the algorithm for calculating ∆τ, see [6].

As a consequence, it is precisely at large values of the l/d parameter that the patterns of changes in the strength of the composite and the adhesive strength in its elementary unit should be compared. In Figure 8, such a comparison was carried out for two unidirectional fiberglass plastics. The correlation curves σ_t_–τ (Figure 8c) for these materials are increasing straight lines. In these fiberglass plastics, the adhesive strength had not reached “ideal” values, at which the strength of the material does not depend on the adhesion strength of its component. This dangerous defect, from which the destruction of the entire material begins, is located at the “fiber–matrix” interface; therefore, to increase the strength of fiberglass plastics, it is necessary to improve the adhesion strength of the binder to the fiber. Correlation curves like these are useful both for those who create composites and for those who use them.

## 5. Conclusions

Studies of the adhesive strength τ of polymer–fiber systems with a wide variation of joint areas S (l/d) made it possible to show how residual stresses τ_res_ present at the interface affect the values of τ measured by the pull-out method, as well as the shape of curves describing the dependence of τ on the studied factor.

It turned out that as a result of the action of τ_res_, the adhesive strength for the same pair depended on the joint areas. The scale dependence of τ–S was described by a monotonically decreasing curve. The change in τ values during the curing of the “fiber–thermoset” systems was described by a curve with a maximum for joints with ratio l/d > 7–10 and a curve with saturation at l/d < 4.

The temperature dependence of adhesive strength over a wide temperature range could be described by three types of curves. If l/d < 4, τ values continuously decreased while T increased. When l/d ≤ 4–7, adhesive strength during the temperature growth stayed constant and then decreased. The τ change in joints with the ratio l/d > 10–15 during the temperature growth was more complex. In this case, adhesive strength grew to a certain temperature, and then it decreases.

This observed ambiguity should be taken into account when discussing the influence of the interface on the properties of composites and their fracture mechanism (for example, when constructing “composite strength–adhesive strength” correlation curves, and for identification of the “weak” link in composites and localization of a possible source of their fracture). In this case, the Fvalues of adhesive systems with l/d ≥ 10–15 should be used.

The analysis carried out and the obtained patterns are valid for systems in which the strength of the interface is ensured by strong (chemical) interactions (for example, during the interaction of epoxy resins with fibers that have high surface energy, such as glass, boron, steel, and basalt fibers).

The obtained and summarized results allow us to expand our understanding of residual stresses arising at the polymer–fiber interface and make a significant contribution to the understanding of the implementation of fiber strength in reinforced plastics.

## Figures and Tables

**Table 1:** Compositions of the systems presented in Figure 2.

Binder	Binder Type	Curing Conditions	Glass Temperature	Fibers
EDT-10	Amine	160 °C—8 h	106 °C	Steel
EAnhB	Anhydride	90 °C—3 h, 120 °C—12 h	121 °C	Steel
MAB	Amine	160 °C—8 h	80 °C	Glass
PPRS	Polyphenilsiloxane	200 °C—6 h	130 °C	Glass

**Table 2:** Measuring ranges for the parameters of the systems presented in Figure 2.

Binder	Fibers	Length l, mm	Area S, mm^2^	l/d
EDT-10	Steel	0.3–3.1	0.158–1.45	2–20.5
EAnhB	Steel	0.6–2.1	0.27–1.01	4–14
EDT-10	Glass	0.15–0.32	(5.5–11.5)∙10^−3^	13–28
PPRS	Glass	0.15–0.35	(5.5–12.5)∙10^−3^	13–30

**Table 3:** T_max_ value on the dependences τ–T of “thermosetting binder–alkali-free glass fiber” compounds, curing modes, and glass transition temperatures of binders T_g_.

Binder	T_g_, °C	T_max_, °C	Curing Conditions
BP-4	65	~30	70 °C—2 h, 90 °C—2 h, 110 °C—1.5 h, 130°C—1.5 h, 160 °C—2 h
PPSR	130	30	200 °C—6 h
PN-1	30	~90	100 °C—6 h

## Data Availability

Data are contained within the article.
